# Squeezing Mechanical Analysis and Model Establishment of the Viscoelastic Rubber-Strip-Feeding Process of the Cold-Feed Rubber Extruder

**DOI:** 10.3390/polym14173602

**Published:** 2022-08-31

**Authors:** Yanchang Liu, Yiren Pan, Xuehua Hu, Fang Yu

**Affiliations:** College of Electromechanical Engineering, Qingdao University of Science and Technology, Qingdao 266061, China

**Keywords:** squeezing mechanical analysis, rubber strip cold-feed process, viscoelastic rubber strip

## Abstract

In the process of rubber extrusion, the feed structure directly affects the extrusion quality, extrusion uniformity, screw lateral force, and feed power consumption. Until now, the feed structure was mainly based on empirical designs, and there was no theoretical model for the optimal design of a feed structure. This paper focused on the squeezing mechanical analysis and model establishment of the feeding process in which viscoelastic rubber strips are passed through feed-wedge clearance in cold-feed extruders. The screw flight rotation squeezing process was simplified into a disc rotation squeezing process; the instantaneous squeezing velocity $\dot{h}\left(t\right)$ in the disc rotation squeezing model was derived according to feed wedge clearance geometry and the disc rotating speed. By transforming rotation squeezing into differential slab squeezing, mathematical expressions of the velocity distribution, pressure distribution, total squeezing force, and power consumption in the feeding process were derived in a rectangular coordinate system under isothermal and quasi-steady assumptions and certain boundary conditions by using balance equations and a Newtonian viscous constitutive relation. Theoretical calculations and experimental values showed the same trend. Through comparison, it was found that the power consumption (P3) caused by sliding friction is about 200–900 W according to theoretical calculations, while the experimental test results show it to be about 300–700 W. Additionally, the difference between theoretical pressure value and the experimental pressure value can be controlled within 5–15%. This could reflect the main factors that affect the feeding process, so could be used for analyses of actual feeding problems, and to contribute to rough quantitative descriptions of the feeding process, finite element simulation, and the optimization of the feeding structure.

## 1. Introduction

**Figure 1:**
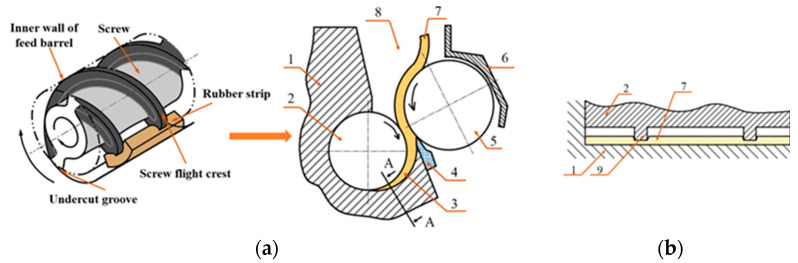
(**a**) Feed wedge clearance; (**b**) A-A section view; Feeding model of a cold-feed rubber extruder. (1) Feed barrel; (2) screw; (3) feed wedge clearance; (4) scraper; (5) feed roll; (6) feed door; (7) rubber strip; (8) feed opening; (9) screw flight.

The extrusion process is an important step in rubber processing, and the vast majority of rubber compounds are extruded at least once during the molding process [1]. The feeding process has a significant impact on extrusion quality [2,3], extrusion stability, the side force applied to screw, and extruder power consumption. In modern research and applications, in order to realize a uniform feed rate in rubber extruders, rubber strip feeding has been used in either rarely used hot-feed extruders or widely used cold-feed extruders. Because rubber has relatively strong adhesiveness at room temperature in a highly elastic state, the cost of the granulation process will increase if a rubber extruder is fed granulated material. A large number of separating agents can be added to prevent the adhesion phenomenon of rubber granulates, resulting in changes in the formulation properties [4]. For a cold-feed extruder fed with a rubber strip form, regardless of whether a free-feeding structure or the widely used forced-feeding structure with a feed roll is used, the feed wedge clearance is always designed at the feed port. Figure 1a shows the common feeding model (feed roll structure) of a cold-feed rubber extruder. The feed wedge clearance is formed between the screw flight crest and the undercut groove on the inner wall of the feed barrel. The feed wedge clearance is a key structure when feeding the rubber strip into the screw channel: the rubber strip is gradually squeezed (compressed) and thinned when the rotating screw flight drags the rubber strip through the feed wedge clearance. At the same time, the screw flight is more deeply embedded into the rubber strip (Figure 1b). When the rubber strip approaches the minimum wedge gap (the design gap between the outer diameter of the screw and the inner wall of the feed barrel), it is longitudinally broken off [5], thus entering the channels at both sides of the screw flight and completing the feeding process of the rubber strip. In a combined structure of a feed roll/screw system and the feed wedge clearance, the feed roll only increases the pushing force [6] when feeding the rubber strip or creating an additional pressure build-up [7]. Therefore, when using rubber extruders, the rubber strip is dragged through the feed wedge clearance and broken off longitudinally at the end of the feed wedge clearance; this is the key to successfully carrying out rubber strip feeding. In the vicinity of the minimum wedge gap, the thin strip easily causes longitudinal break-off under the axial thrust action of the screw flight. Since rubber is essentially a viscoelastic liquid at room temperature [8], a special kind of squeezing (compression) flow occurs in the feed wedge clearance. The feed wedge clearance geometry is a nonlinear wedge region with a narrow width (the width of the screw flight). The squeezing force is generated by dynamic viscous pressurization in the nonlinear wedge region through the rotation drag motion of the screw flight, and rubber flows in the nonlinear wedge region occur only in the directions towards both sides of the screw flight, because the minimum wedge gap is very small, and can be neglected for rubber compounds with very high viscosity.

The history of rubber extruders dates back more than 140 years, to the invention of hot-feed extruders in 1879. The continuous development of feeding technology is mainly reflected in the improvements to the feeding structure [9,10,11,12,13] and the control of feeding uniformity [4,14,15,16]. However, there has been little research into the feeding theory of rubber extruders, especially cold-feed extruders. The existing research on the theory of the rubber-strip feeding process has either analyzed the mechanical conditions of rubber strips being drawn into the feed wedge clearance [6], or used a disc rotation squeezing model device to carry out experimental research on the strip feeding process [5]. Since the study by Jianbin Li did not consider the contribution of the flight flanks to the additional drag effect [6], it reached the incorrect conclusion that the feeding mechanical condition of the friction coefficient μ_s_ between the flight crest and the rubber strip was greater than the friction coefficient μ_b_ between the barrel inner wall and rubber strip. Other experimental results in the studies of Yanchang Liu [5] included single-peak pressure distribution and the obvious power consumption when the rubber strip passed through the model clearance. To date, little theory of the rubber strip feeding process exists, which makes the feeding theory for rubber extruders lag far behind engineering practice. The side force applied to the screw and the feed power consumption have not been calculated theoretically, and the feed structure and technology have not been improved or innovated by theoretical guidance.

In order to establish a feeding process theory of cold-feed rubber extruders, the squeezing flow dynamics during this process must be analyzed. However, most studies of squeezing flow deal with the pure squeezing flow of viscoelastic materials [17,18,19,20,21,22,23,24,25,26,27,28], or viscoelastic materials [29,30,31,32,33,34,35,36,37,38,39,40], or non-Newtonian liquids with a squeezing–extrusion combined flow [41] between two parallel plates; very little research has considered the squeezing flow between two non-parallel plates. The squeezing and sliding flow of Oldroyd-B fluid was examined by N. Phan-Thien [42] in a wedge geometry of semi-wedge angle *a*(*t*) with a wedge apex, using a numerical method. In this wedge, the flat plane boundaries of the wedge were closing at a rate $\dot{a}\left(t\right)$ and were sliding along the direction perpendicular to the two-dimensional wedge with a constant velocity. However, there was no consideration of the squeezing flow of non-Newtonian liquids in a nonlinear wedge region, in which the lubrication approximation was not valid due to large wedge angle or slip boundary condition. The squeezing flow in feed wedge clearance belongs to the latter category, because the rubber strip slides along the cylinder wall in the feeding process. Therefore, in this study, we attempted to analyze the dynamics of squeezing flow caused by the drag action of the screw flights in the nonlinear wedge region under sliding boundary conditions. In order to obtain an approximate analytical solution of the motion equation which reflects the basic characteristics of the squeezing flow, under some assumptions (Newton material, isothermal quasi-steady flow), the physical model was simplified (disc rotation squeezing model) and the motion transformation (differential slab squeezing flow) was applied. To verify the accuracy of the theoretical model, a comparison between the theoretical calculations and the experimental data was carried out. The theoretical model that was established could provide guidance and suggestions for the design and optimization of actual feed structures.

## 2. Physical Model and Squeezing Velocity

In this section, a physical model of feeding process is established and the calculation formula of squeezing velocity in the feed wedge clearance is obtained by analyzing the relationship between the rotation angle and the wedge gap.

### 2.1. Physical Model

**Figure 2:**
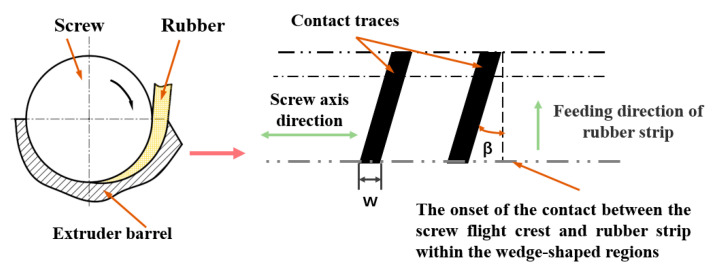
Expanded view of the contact trace between the screw flight crest and the rubber strip.

**Figure 3:**
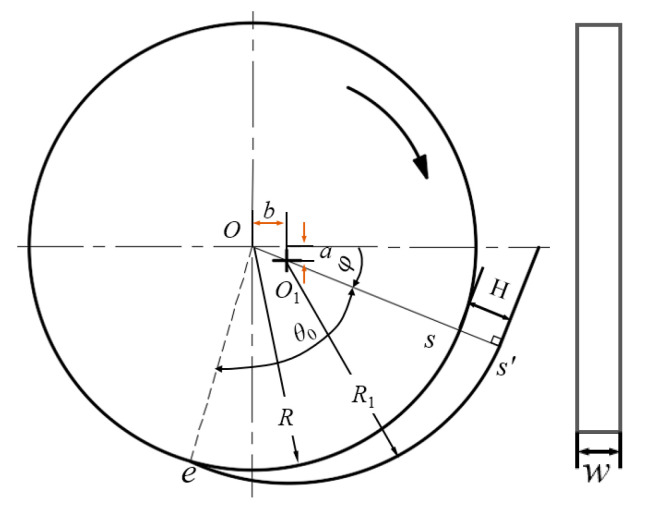
Disc rotation squeezing model.

At the feed port, the basic screw configuration of the cold feed extruder is a single screw section with double-flighted design. When rubber strips pass through the feed wedge clearance, the screw flight crest will always contact and squeeze the rubber strip. If expanded along the outer diameter of the screw, the contact traces between the screw flight crest and the rubber strip are two inclined narrow strips (the black areas in Figure 2). In Figure 2, *β* is the helical angle and *w* is the axial width of the flight. To describe the behavior of the feeding process, the geometry of the helix feed wedge clearance is simplified, and then motion transformation is applied, as discussed in Section 3.1. As the rubber strip passes through the feed wedge clearance, the effective flight compression path is perpendicular to the screw axis. Therefore, each feed’s screw flight can be simplified to a disc (helical angle *β* = 0°), and the diameter and width of the disc are screw diameter *D* and screw flight axial width *w*, respectively. An effective feed wedge clearance is formed between the disc and the undercut groove on the inner wall of the feed barrel. In this way, a disc rotation squeezing model is built with a disc and a barrel (Figure 3). Because the two screw flights have the same geometry, one was chosen for mathematical description.

For all general specifications of cold-feed extruders, a standardized design of the feed wedge clearance has been achieved. In Figure 3, *a* = 0.03 − 0.04*D*, *b* = 0.06 − 0.08*D*, *R* = 0.5*D*, *R*_1_ ≈ 0.5*D*, H is the start position or maximum value of the wedge gap (on the extended line of *O* and *O*_1_, that is $H=\bar{ss^{\prime }}$), $h_{\min}$ is the end position or minimum value of the wedge gap (the typical value is 0.0045*R*), and $\theta_{0}$ is the center angle of the circular arc of the outer diameter of the disc in the whole range of the feed wedge clearance. For example, for a cold-feed extruder with *D* = 65 mm, *a* = 2 mm, *b* = 5 mm, *R* = *R*_1_ = 32.5 mm, *H* = 5.4 mm, *h*_min_ = 0.146 mm, and *θ*_0_ = 85° ≈ 1.483 rad.

### 2.2. Squeezing Velocity

**Figure 4:**
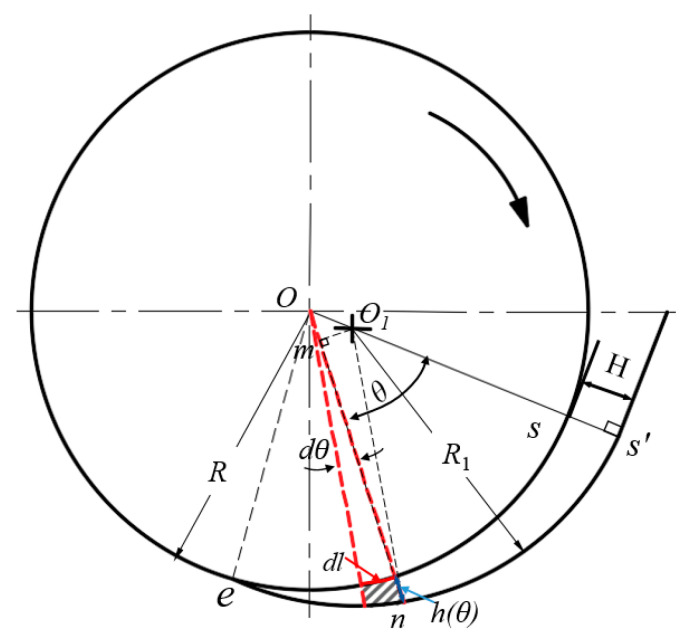
Relationship between the gap h(θ) and the angle θ.

An analysis of squeezing flow needs to determine either the squeezing force or the squeezing velocity. In the disc rotation squeezing model, the squeezing force is unknown, and the squeezing velocity can be derived according to the geometry of the feed wedge clearance and the disc speed. Figure 4 shows the relationship between the gap h(θ) and the angle θ.

If the initial thickness of the fed rubber strip is equal to the maximum value *H* of the wedge gap, the position of the line segment *ss* can be set as the initial point at which the rubber strip enters into the wedge clearance. When the disc rotates through an angle *θ* (rad) from time *t* = 0 to *t*(s), the strip is squeezed (compressed) from thickness *H* to *h*(θ) (Figure 4). The mathematical relationship between *h*(*θ*) and *θ* is

(1) $$h(\theta )=(\bar{Om}+\bar{mn})-R=c\cos\theta +\sqrt{R_{1}^{2}-{\left(c\sin\theta \right)}^{2}}-R$$

where $c=\bar{OO_{1}}=\sqrt{a^{2}+b^{2}}$.

By expanding the term with the root sign on the right side of Equation (1) into a power series,

(2) $$\sqrt{R_{1}^{2}-{\left(c\sin\theta \right)}^{2}}=R_{1}\left[1-\frac{1}{2}\frac{{\left(c\sin\theta \right)}^{2}}{R_{1}{}^{2}}+\ldots \right]$$

By ignoring the terms greater than the quadratic term of the power series on the right side of Equation (2) and substituting it into Equation (1) and considering *R* ≈ *R*_1_, one obtains

(3) $$h(\theta )=ccos\theta -\frac{c^{2}}{2R_{1}}sin^{2}\;\theta$$

If the rotating speed of the disc is *N*rpm, then *θ* = *πNt*/30. Substituting the expression of *θ* into Equation (3) gives

(4) $$h(\theta )=ccos\left(\frac{\pi Nt}{30}\right)-\frac{c^{2}}{2R_{1}}sin^{2}\;\left(\frac{\pi Nt}{30}\right)$$

By differentiating both sides of Equation (4), one can obtain the instantaneous squeezing velocity $\dot{h}\left(t\right)$,

(5) $$\dot{h}(t)=-\frac{\pi Nc}{30}\left[\sin(\frac{\pi N}{30}t)+\frac{c}{2R_{1}}\sin(\frac{\pi N}{15}t)\right]$$

where “−” indicates squeezing (compression). From Equation (5), $\dot{h}$ is directly related to the geometric parameters of the feed wedge gap clearance (*c* and *R*_1_) and the operating parameters (*N* and *t*).

**Figure 5:**
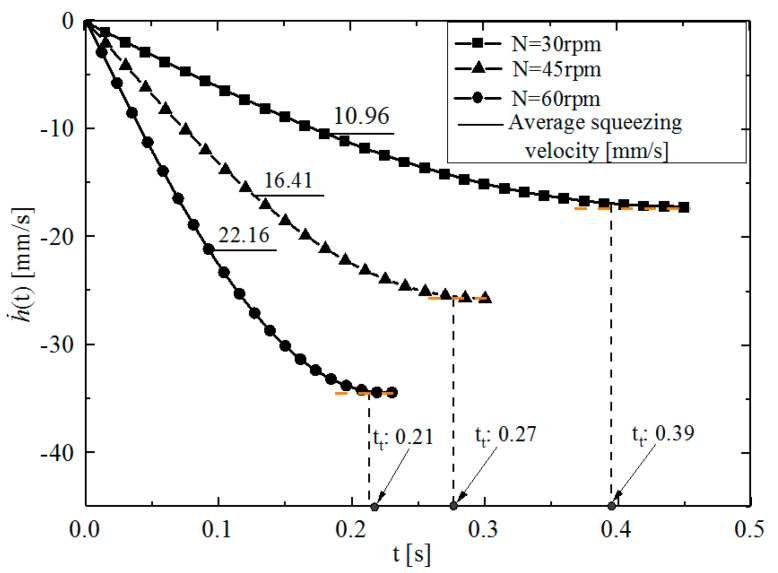
$\dot{h}-t$ curves in the disc rotation squeezing model with *D* = 65 mm at different disc speeds.

To study the change in instantaneous squeezing velocity, according to Equation (5), MATLAB software was used to calculate and draw $\dot{h}-t$ curves at different disc speeds (*N* = 30, 45 and 60 rpm) for the disc rotation squeezing model of *D* = 65 mm ($c=\sqrt{2^{2}+5^{2}}\approx 5.39\;\mathrm{mm}$, *R*_1_ = 32.5 mm). Figure 5 shows that the $\dot{h}-t$ curve is approximately composed of two parts: a constant acceleration squeeze in the early stage and a constant velocity squeeze in the later stage. The former accounts for most of the whole rotational squeezing process, and the latter accounts for a small part. Setting *t*_0_ and *t*_t_ to represent the time used during the whole rotational squeezing process and the transition time from the constant acceleration squeeze to the constant velocity squeeze, respectively, *t*_0_ can be calculated according to the formula *t*_0_ = 30*θ*_0_/*πN*, and *t*_t_ can be measured from the asymptotic transition part of the $\dot{h}-t$ curve in Figure 5. When *N* = 30, 45, and 60 rpm, *t*_0_ is 0.47, 0.31, and 0.24 s, *t*_t_ is 0.39, 0.27, and 0.21 s, and *t*_t_/ *t*_0_ is 0.83, 0.87, and 0.875, respectively. Figure 5 also shows that the squeezing velocity increases faster with increasing disc speed. Similar results can be obtained by using the same method to calculate other disc rotation squeezing models with different diameters (*D* = 90, 120, 150, 200, and 250 mm).

The above analysis shows that, for a given disk rotating squeezing model and disc rotating speed, the instantaneous squeezing velocity $\dot{h}\left(t\right)$ changes rapidly for most of the time taken for the whole squeezing process. This presents difficulties when analyzing the squeezing flow. To simplify the theoretical analysis, the average squeezing velocity (−*V*) was introduced, where ‘−’ also represents the squeezing direction, V=|−V|. According to *N*, *H*, and *θ*_0_, it is easy to calculate *V*.

(6) $$V=\left|-V\right|=\left|-\frac{H}{t_{0}}\right|=\left|-\frac{\pi NH}{30\theta_{0}}\right|=\frac{\pi NH}{30\theta_{0}}$$

Equation (6) shows that, for a given disc rotation squeezing model, the average squeezing velocity *V* is only proportional to *N*. For example, for the disc rotation squeezing model with *D* = 65 mm, the average squeezing velocities calculated by Equation (6) at *N* = 30, 45, and 60 rpm are −11.4, −17.1, and −22.8 mms^−1^, respectively. For the disc rotation squeezing models for other general specifications of cold-feed extruders, when the rotational speed *N* = 30 rpm, the average velocities calculated by Equations (5) and (6) in the whole feed wedge gap are *V*_(5)_ and *V*_(6)_, respectively. The range of [(*V*_(5)_ − *V*_(6)_)/*V*_(5)_] × 100% is approximately −0.51~1.30%, which strongly indicates that the simplified Equation (5) has a very high level of accuracy.

## 3. Mathematical Model

The disc rotation squeezing model established in Section 2 was further simplified into a differential slab squeezing model. According to the balance equations, mathematical formulas including velocity, pressure distribution, total squeezing force, and power consumption in the wedge clearance were obtained.

### 3.1. Kinematic Exchange

**Figure 6:**
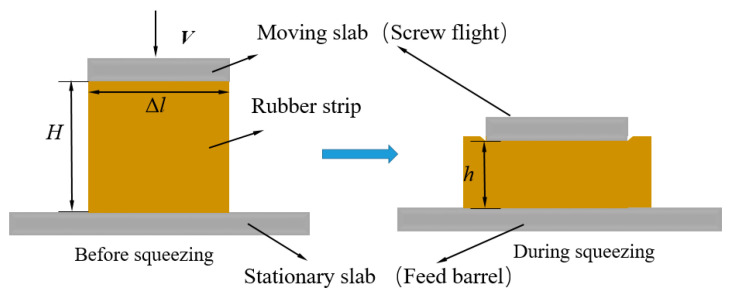
Differential slab squeezing flow.

During the actual feeding process, the rubber strip that is adhered to the screw flight rotates with the rotational screw flight and slides along the barrel surface. Therefore, in the disc rotation squeezing model, the disc drags the rubber strip to rotate through an angle *θ* (rad) within time *t*, and the rubber strip is squeezed (compressed) from the initial thickness *H* to the thickness *h*(t). This process can be regarded as one in which a “differential slab” of arc length Δl (Figure 4) squeezes the strip in parallel from the initial thickness *H* to the thickness *h*(t) within time *t* at the squeezing velocity generated by the disc rotation. The squeezing flow obtained from the kinematic exchange is called the differential slab squeezing flow, which is shown in Figure 6. Since screw flight is always embedded into the rubber strip during the feeding process, this flow belongs to a constant area squeezing flow [43].

### 3.2. Velocity and Pressure Distributions

**Figure 7:**
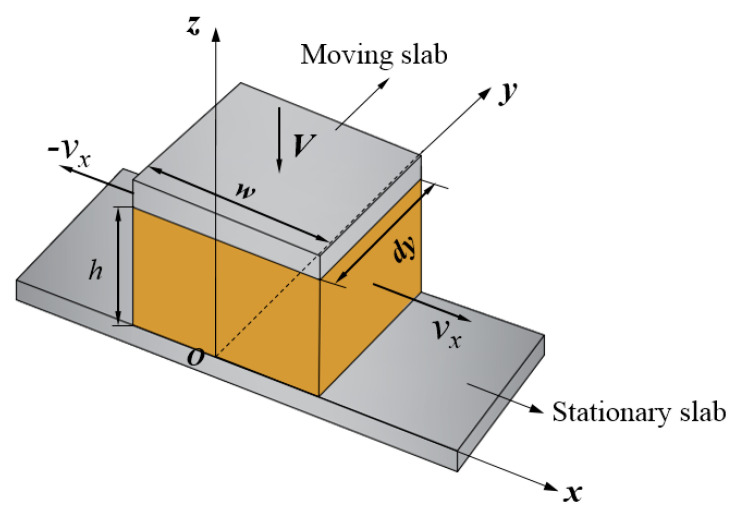
The rectangular coordinate systems used in the theoretical analysis, dy=Δl.

The theoretical analysis used the rectangular coordinate system shown in Figure 7. First, the following assumptions about the differential slab squeezing flow were made to solve the balance equations for the analytic solutions of the velocity and pressure distributions:

(i) The flow of the rubber compound is isothermal, has a quasi-steady state [42,44], and is laminar. In the quasi-steady state, locally and instantaneously, the squeezing flow may be regarded as a steady flow between fixed parallel plates [45].

(ii) The inertia, gravity, and normal stress terms in the momentum equation can be ignored.

(iii) The rubber compound is a noncompressible Newtonian liquid.

To simplify the analysis of the parallel plate squeezing flow, the component of momentum in the direction parallel to the plates (here, it is the *x*-component of momentum) is usually assumed to be the most important, and the component of momentum in the direction normal to the plates (here, it is the *z*-component of momentum) is not used [45]; that is, the component of velocity $v_{z}$ normal to the plates is neglected [46].

Because the minimum value *h_min_* of the feed wedge gap is very small (*h_min_* ≈ 0) and there is an additional pressure build-up created by the feed roll/screw system in the upstream position of the maximum wedge gap, the component of velocity $v_{y}$ in the direction of the differential dimension (*y*-direction) can be assumed to be zero in the differential slab squeezing flow. In other words, the rubber compound can only be squeezed out along the directions on both sides of the disc (or flight) (*x*-direction) during the feeding process. This is an important boundary condition for the disc rotation squeezing model or the differential slab squeezing flow.

Based on the above assumptions and analyses, and considering the isotropic pressure and the component of velocity to be constant at the differential dimension *dy,* the equations of momentum can be reduced to a one-dimensional form as follows:

(7) $$\eta \frac{\partial^{2}v_{x}}{\partial z^{2}}=\frac{\partial p}{\partial x}$$

where *v_x_* is the x-component of velocity and *p* is isotropic pressure.

Because the rubber strip slides along the barrel surface during the feeding process, it is assumed that there is no slippage at the moving slab (screw flight crest) and at the stationary slab of the x-direction, that is, the boundary condition *z* = 0, *v_x_* = 0 and *z = h*, *v_x_* = 0. Integrating Equation (7) twice with respect to *z* and applying the boundary conditions can derive Equation (8):

(8) $$v_{x}=\frac{1}{2\eta }\frac{\partial p}{\partial x}(z^{2}-hz)$$

This *v_x_* velocity distribution must satisfy the following overall continuity relationship:

(9) $$Vxdy=\int_{0}^{h}v_{x}dydz$$

By substituting Equation (9) into Equation (8) and considering *dy* as a constant value:

(10) $$\frac{\partial p}{\partial x}=-\frac{12\eta V}{h^{3}}x$$

Usually, the screw channel at the feed port is not fully filled with rubber [5]. Therefore, it can be assumed that the pressure on both sides of the disc (screw flight) is atmospheric pressure, that is, x=±w/2, p=0. Under this boundary condition, integrating Equation (10) can obtain:

(11) $$p=\frac{3\eta Vw^{2}}{2h^{3}}-\frac{6\eta V}{h^{3}}x^{2}$$

**Figure 8:**
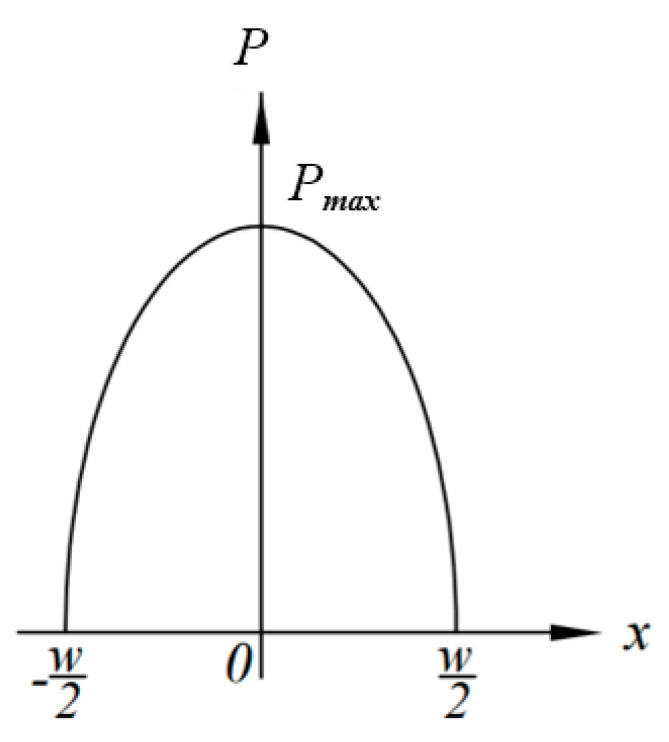
Relationship between *p* and *x*.

Equation (11) shows that the pressure distribution *p* in the model wedge *p* is related to *V*, *w*, *h*, *x*, and *η*, and the effects of *h*, *x*, and *w* on *p* are particularly significant. For a given disc rotation squeezing model, when the disc rotating speed *N* is constant, *p* is only related to *x* at any squeezing thickness *h*, showing the parabolic distribution shown in Figure 8. Figure 8 shows that, for an arbitrary *h*, the maximum pressure *p_max_* can be obtained at *x = 0*.

According to Equation (11), it is easy to calculate the average pressure $\bar{p}$ along *w* in Figure 8:

(12) $$\bar{p}=\frac{\int_{-\frac{w}{2}}^{\frac{w}{2}}\left(\frac{3\eta Vw^{2}}{2h^{3}}-\frac{6\eta Vx^{2}}{h^{3}}\right)dxdy}{wdy}=\frac{\eta Vw^{2}}{h^{3}}$$

Equation (12) shows that, for a given disc rotation squeezing model and rubber compound, when the disc rotation speed is constant, $\bar{p}$ is inversely proportional to the cubic power *h*, proportional to the quadratic power *w*, and proportional to *η*. As *h* decreases, $\bar{p}$ increases rapidly, and when h→0, $\bar{p}\to \infty$. However, p or $\bar{p}$ is always a finite value because h has a minimum design value *h_min_* (at *θ = θ*_0_). In addition, Liu Y. C. and Yu F [3] used a disc rotation squeezing model device with *D* = 65 mm and found that the rubber strip would be longitudinally broken off at a position of approximately *h* = 1 mm before the design value of the minimum wedge gap *h_min_*(=0.16 mm). *h_b_* and *θ*_b_ are set as the wedge gap value and the corresponding center angle when the rubber strip is longitudinally broken off, respectively. For the disc rotation squeezing model with *D* = 65 mm and *h_b_* = 1 mm, the *θ*_b_ = 73° can be obtained by calculating or drawing, but the regularity of *h_b_* and *θ*_b_ needs further experimental research.

A combination of Equations (8) and (10) obtains:

(13) $$v_{x}=-\frac{6Vx}{h^{3}}(z^{2}-hz)$$

From Equation (13), *v_x_* depends on *V*, *h*, *x*, and *z*. When *V*, *h*, and *x* are fixed, the relationship between *v_x_* and *z* shows a parabolic curve, and shear flow occurs along the z-direction. When *V*, *h*, and *z* are fixed, *v_x_* is proportional to *x* and produces elongational flow in the *x* direction. When *V*, *x*, and *z* are fixed, *v_x_* is inversely proportional to the cubic power of *h*; that is, when *h* decreases, *v_x_* increases rapidly.

### 3.3. Total Squeezing Force

The total squeezing force refers to the resultant force of the pressures exerted on the rubber strip in the radial direction of the disc (screw) within the whole feed wedge clearance. In the design of a rubber extruder, for example, the total squeezing pressure is used to calculate the elastic flex deformation of the screw caused by the side force, because excessive flex deformation will cause scraping between the screw and the barrel.

**Figure 9:**
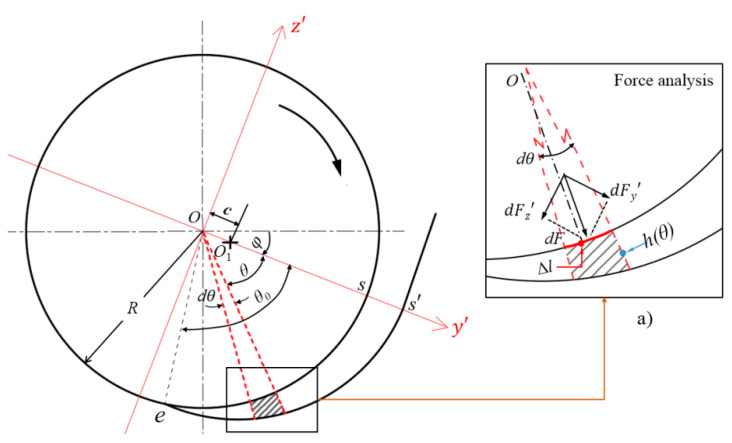
Force analysis of the disc during squeezing; (**a**) enlarged drawing of the force analysis.

The total squeezing force F can be deduced from the disc rotation squeezing model. The method for calculating F is as follows: first, the coordinate system $y^{\prime }Oz^{\prime }$ shown in Figure 9 was established, and the force dF acting on the area element wdl(=wRdθ) at position θ in the radial direction of the disc was decomposed into $dF_{\;y^{\prime }}$ and $dF_{\;z{}^{\prime }}$ components, as shown in the enlarged view. Second, the magnitudes of the $F_{\;y{}^{\prime }}$ and $F_{\;z{}^{\prime }}$ components of the total squeezing force F were calculated by integral, that is, $F_{\;y{}^{\prime }}=\sum^{\;}\left|dF_{\;y{}^{\prime }}\right|$ and $F_{\;z{}^{\prime }}=\sum^{\;}\left|dF_{\;z{}^{\prime }}\right|$. Finally, the magnitude and tangential direction angle of the total squeezing force F were calculated, that is, $F=\sqrt{F_{\;y{}^{\prime }}{}^{2}+F_{\;z{}^{\prime }}{}^{2}}$ and $\tan\psi =F_{\;z{}^{\prime }}∕F_{\;y{}^{\prime }}$. φ+arctanψ is the angle between the direction of the total squeezing force and the horizontal line.

As mentioned in Equation (7), the pressure in the model wedge is isotropic. Therefore, the pressure in the radial direction in Figure 9 is equal to the pressures calculated by Equations (11) and (12). To simplify the calculation, the average pressure $\bar{p}$ was used. Hence:

(14) $$dFy^{\prime }=\bar{p}wR\cos\theta d\theta$$

(15) $$dFz^{\prime }=\bar{p}wR\sin\theta d\theta$$

Putting Equation (12) into Equations (14) and (15) and integrating them obtains:

(16) $$Fy{}^{\prime }=\int_{0}^{\theta_{b}}\bar{p}w\cos\theta Rd\theta =-\frac{\eta \pi NHw^{3}R}{30\theta_{0}c^{3}}k_{1}$$

(17) $$Fz^{\prime }=\int_{0}^{\theta_{b}}\bar{p}w\sin\theta Rd\theta =-\frac{\eta \pi NHw^{3}R}{60\theta_{0}c^{3}}k_{2}$$

where $k_{1}=tan\theta_{b}$, $k_{2}=\left[{\left(1-\frac{\theta_{b}{}^{2}}{2}+\frac{\theta_{b}{}^{4}}{4!}\right)}^{-2}-1\right]$.

Substituting Equations (16) and (17) into the above expressions of *F* and tanψ, respectively, can obtain:

(18) $$F=\sqrt{{\left(Fy{}^{\prime }\right)}^{2}+{\left(Fz{}^{\prime }\right)}^{2}}=\frac{\eta \pi NHw^{3}R}{60\theta_{0}c^{3}}\sqrt{4k_{1}{}^{2}+k_{2}{}^{2}}$$

(19) $$\tan(\psi )=\frac{Fz^{\prime }}{Fy^{\prime }}=\frac{k_{1}}{2k_{2}}$$

For each given model wedge, *φ* is known; therefore, according to Equation (19), the angle φ+arctanψ between the direction of the total squeezing force and the horizontal line can be obtained.

### 3.4. Power Consumption

When the rubber strip passes through the model wedge, the power consumption comes from the following three parts:

(i) The increase in kinetic energy when the rubber compound is squeezed out from both sides of the disc (screw flight);

(ii) Viscous dissipation in squeezing flow;

(iii) Friction loss of the rubber strip sliding along the barrel.

(1) Power consumption $P_{1}$ caused by an increase in kinetic energy

As h decreases, $v_{x}$ shows a rapid increasing trend (Equation (13)), which increases the kinetic energy of the rubber in the extrusion direction (x direction); the kinetic energy reaches its maximum at the edges at both sides of the disc (screw flight). In Figure 7, the volumetric flow rate in the direction of +x or −x caused by the moving slab squeezing downwards is Vxdy (*V* is the average squeezing velocity, which is shown in Equation (6)). Therefore, the average extrusion velocity ${\bar{v}}_{x}$ at the x position is:

(20) $${\bar{v}}_{x}=\frac{Vxdy}{hdy}=\frac{Vx}{h}$$

If the flow between the approaching slabs is regarded as potential flow, the average velocity ${\bar{v}}_{x}$ in the x direction is a function of x, not h [16]. The average velocity of the rubber extruded from the edges of the slabs (*x = ±w/*2) is ${\bar{v}}_{w∕2}=Vw/2h$ (only the magnitude of the velocity considered). Therefore, the rate of work performed on a moving slab with *dy* length elements is equal to the rate of change of the kinetic energy of rubber *dP*_1_.

(21) $$dP_{1}=\frac{d}{dt}(\frac{1}{2}m{\bar{v}}_{\frac{w}{2}}{}^{2})=\frac{V^{2}w^{2}}{8h^{2}}\frac{dm}{dt}$$

where dm/dt is the mass flow rate of the extruded rubber, which is equal to 2hdy(Vw/2h)ρ, and ρ is the density of rubber, so Equation (21) becomes:

(22) $$dP_{1}=\frac{\rho V^{3}w^{3}}{8h^{2}}dy$$

According to Figure 2, considering dy=Rdθ and integrating Equation (22) along the whole wedge gap yields *P*_1_:

(23) $$P_{1}=\int_{0}^{\theta_{b}}\frac{\rho V^{3}w^{3}}{8h^{2}}Rd\theta$$

Considering the expression of h(θ) (Equation (3)) will make the integral operation of Equation (23) very complex. Therefore, the simple arithmetic mean *H/2* of h(θ) was used to replace h(θ) to obtain the simple approximate solution of *P*_1_:

(24) $$P_{1}=\frac{R\theta_{b}\rho V^{3}w^{3}}{2H^{2}}$$

(2) Power consumption $P_{2}$ caused by viscous dissipation.

The velocity gradient of $v_{x}$ in the *z* direction (Equation (13)) will cause viscous heat generation. Therefore, the shear rate $\dot{\gamma }$ can be obtained by differentiating Equation (13) with respect to *z*:

(25) $$\dot{\gamma }=\frac{\partial v_{x}}{\partial z}=-\frac{6Vx}{h^{3}}(2z-h)$$

From Equation (25): 1) at x=0,$\;\dot{\gamma \;}=0$; 2) at x=±w∕2 and z=0 or h, the absolute value of the $\dot{\gamma \;}$ can take the maximum, ${\left|\dot{\gamma \;}\right|}_{\max}=3Vw∕h^{2}$. To simplify the calculation, the corresponding shear rate at h=H∕2 can be taken as the average shear rate $\bar{\dot{\gamma }}$ in the whole squeezing flow process: that is, $\bar{\dot{\gamma }}=12Vw∕H^{2}$. Therefore, the rate of viscous dissipation of rubber compound per unit volume is:

(26) $$\eta {\bar{\dot{\gamma }}}^{2}=\frac{144\eta V^{2}w^{2}}{H^{4}}$$

If the volume of the whole model wedge is *V*_w_ and the very small volume remaining after the longitudinal break-off of rubber strip is ignored, the power consumption $P_{2}$ caused by viscous dissipation is:

(27) $$P_{2}=\frac{144\eta V^{2}w^{2}Vw}{H^{4}}$$

Through the geometric relationship in Figure 3, *V*_w_ can be obtained:

(28) $$V_{w}=\frac{w(\alpha R_{1}{}^{2}-\theta_{b}R^{2}+Rc\sin\theta_{b})}{2}$$

(3) Power consumption $P_{3}$ caused by sliding friction.

**Figure 10:**
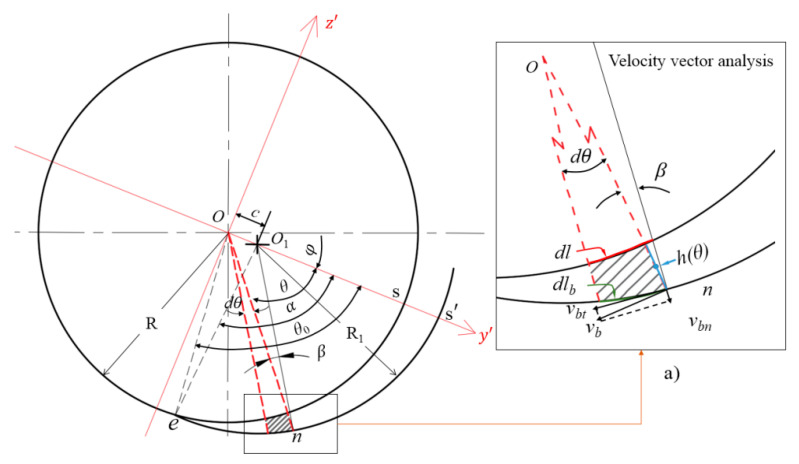
Velocity vector analysis of the rubber strip during squeezing; (**a**) enlarged drawing of velocity vector analysis.

During the sliding process of rubber strips dragged by the disc (screw flight) along the barrel, the shape of the rubber strip is bent, and the local pressure is continuously changed. As shown in Figure 10, for a rubber element at position θ, setting $∠OnO_{1}=\beta$, according to the cosine theorem:

(29) $$\cos\beta =\frac{{\left(R+h\right)}^{2}+R_{1}{}^{2}-c^{2}}{2(R+h)R_{1}}$$

Equation (29) shows that, in the feeding process, the value of β will change with h or θ. Since the center and radius of the cylinder arc $\hat{e\;s{}^{\prime }}$ are $O_{1}$ and $R_{1}$, respectively, the tangential velocity $v_{\mathrm{bt}}$ of the rubber element sliding along the cylinder at position θ is:

(30) $$v_{bt}=v_{b}\cos\beta =\frac{\pi N(R+h)}{30}\cos\beta$$

Obviously, $v_{\mathrm{bt}}$ changes with h or θ.

Assuming that the total squeezing force *F* (Equation (18)) is evenly distributed on arc $\hat{e\;s{}^{\prime }}$, and the sliding friction coefficient *f* between the rubber strip and the barrel is a constant, the friction force $dF_{f}$ generated by the barrel on the rubber element at position *θ* is:

(31) $$dF_{f}=f\frac{F}{\alpha R_{1}}(R+h)d\theta$$

where $\alpha =∠eO_{1}s^{\prime }$ for a given model wedge gap and α is a known constant.

From Equations (30) and (31), the friction power $dP_{3}$ generated by the barrel to the rubber element at position θ can be obtained:

(32) $$dP_{3}=dF_{f}v_{bt}=\frac{\pi NFf}{30\alpha R_{1}}{\left(R+h\right)}^{2}\cos\beta d\theta$$

The power consumption $P_{3}$ caused by sliding friction in the whole feeding wedge is:

(33) $$P_{3}=\frac{f\pi NF\int_{0}^{\theta_{b}}(R+h{)}^{2}\cos\beta d\theta }{30\alpha R_{1}}$$

To simplify the integral operation of Equation (33), the average value $\bar{\beta }$ of β is used, that is, $\bar{\beta }=∠OeO_{1}∕2$. For a given model wedge gap, $∠OeO_{1}$ is known, and $\bar{\beta }$ and $\cos\bar{\beta }$ are known constants. For example, for the disk rotating squeezing model with D=65 mm, $∠OeO_{1}\approx 9.5^{{}^{\circ}}$, $\bar{\beta }=4.75^{{}^{\circ}}$, and $\cos\bar{\beta }\approx 0.9966$. So Equation (34) becomes

(34) $$P_{3}=\frac{f\pi NF\cos\bar{\beta }k_{3}}{30\alpha R_{1}}$$

where

$$\begin{array}{l} k_{3}=\int_{0}^{\theta_{b}}{\left(R+h\right)}^{2}d\theta =R^{2}\theta_{b}+2R\left[c\sin\theta_{b}-\frac{c^{2}}{2R}(\frac{1}{2}\theta_{b}-\frac{1}{4}\sin2\theta_{b})\right]\\ +\left[c^{2}*(\frac{1}{2}\theta_{b}+\frac{1}{4}\sin2\theta_{b})-\frac{c^{3}}{3R_{1}}\sin^{3}\theta_{b}+\frac{c^{4}}{4R_{1}{}^{2}}(\frac{\sin4\theta_{b}}{32}-\frac{\sin2\theta_{b}}{4}+\frac{3\theta_{b}}{8})\right] \end{array}$$

By combining Equations (24), (27) and (34), the power consumption P in the process of the rubber strip passing through the model wedge gap is obtained:

(35) $$P=P_{1}+P_{2}+P_{3}$$

That is:

(36) $$P=\frac{R\theta_{b}\rho V^{3}w^{3}}{2H^{2}}+\frac{144\eta V^{2}w^{3}}{H^{4}}Vw+\frac{f\pi NF\cos\bar{\beta }k_{3}}{30\alpha R_{1}}$$

It should be noted that the above mathematical expressions of total squeezing force and power consumption only consider a single disc. However, for an actual cold feed extruder, there are usually at least double-threaded flights at the feed port, and the calculation of the total squeezing pressure and the power consumption needs to multiply the above expressions by the number of flights.

The theoretical analysis model of the feeding process of cold-feed rubber extruders has important guiding significance for analyzing feeding problems and optimizing and innovating feeding structures: (1) the side force applied to the screw and the feeding power consumption can be roughly calculated without a feed roll, which provides basic calculation parameters for rubber extruder design and overcomes the pure experience of feed structure design. (2) The width of the screw flight at the feed screw segment should be as narrow as possible, because the total squeezing force and power consumption are proportional to the third power of the width of the screw flight. However, a screw width that is too narrow can reduce the drag-in force of the screw flight crest so that the rubber strip cannot be dragged through the feed wedge clearance. For a screw flight with a narrower width, shallow grooves can be opened on the top or the side of the screw flight to increase friction drag action, or else the screw flight can be interrupted to increase “penetration” drag action. The latter is often used in the feed screw segment of modern cold-feed extruders. (3) Under the condition of the same feed wedge clearance length, the maximum value (H) in the feed wedge clearance should be as large as possible, because the total squeezing force and the power consumption are proportional to the reciprocal of the power of H. In other words, a thicker rubber strip should be used for feeding. Therefore, the improvement direction of the structure of the feed wedge clearance was to increase the eccentricity values *a* and *b* and select the appropriate eccentric arc radius *R*. This design can shorten the feeding time and improve the feeding efficiency. (4) Because the power consumption mainly depends on the frictional resistance of the barrel to the rubber strip (see Section 5), the inner wall of the barrel should have a lower friction coefficient at the feed port; that is, the inner wall of the barrel should be smoother than it has been in previous designs. (5) If the feed wedge clearance, feed screw flight, and feed barrel are fully optimized, the effect of feed roll can be reduced or even rendered unnecessary, and the feeding power consumption can be further decreased.

## 4. Materials and Methods

### 4.1. Experiment and Materials

**Figure 11:**
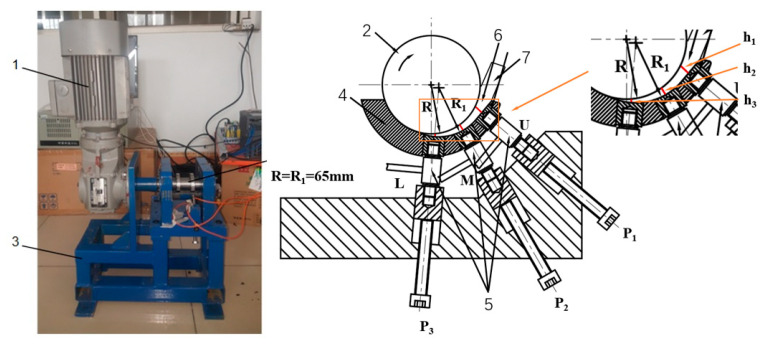
Disc rotation squeezing model device. (1) Motor; (2) disc; (3) bracket; (4) semicylinder; (5) pressure sensor; (6) feed wedge clearance; (7) rubber strip.

The pressure and power data obtained by using the disc rotation squeezing model device (Figure 11) were chosen. Test device: this model device comprises disc 2 driven by motor 1, semicylinder 4 with an eccentric undercut groove on the inner surface, pressure sensor 5, and a control system (which is not shown in the figure). The top surface of the disc, and the inner wall of the groove under the semicylindrical feed wedge clearance 6 and 7, using feeding rubber strips. The disc and drive device are supported by bracket 3. The DJYZ-10 cylindrical pressure sensors were installed in the upper (U), middle (M), and lower (L) positions of the semicylinder, and were used to measure the pressures of the rubber strip through the feed wedge clearance. The pressure sensor had a measurement range of 0–500 kg and a nonlinearity of 0.3–0.5%. The pressure and power data were automatically calculated, recorded, and stored by upper computer software. The disc speed was changed by a variable frequency speed-regulating motor. The temperature of the laboratory was set at 23 ± 2 °C. The geometric parameters of the model device are shown in Table 1.

Test materials: truck radial tire compound, ruck tire inner liner compound (TTI) ((51 ML1 + 4 (100 °C); the Mooney viscosity of the rubber obtained by testing at 100 °C is 51), truck tire sidewall compound (TTS) (55 ML1 + 4 (100 °C)), and truck tire tread compound (TTT) (65 ML1 + 4 (100 °C)), provided by Shandong Anchi Tire Co., Ltd. The size of the feeding rubber strip was approximately 5.4 (thick) × 65 (width) × 100 (length) mm.

Test method: rotation squeezing tests were carried out at room temperature using the above three kinds of rubber compounds to measure the pressure distribution and power consumption of the feed wedge clearance under different disc speeds (30, 45, and 60 rpm).

### 4.2. Measurements of Viscosity and the Sliding Friction Coefficient

The shear viscosity and sliding friction coefficient of the above three compounds at room temperature are two physical parameters required by the theoretical model. These two parameters must be measured.

(1) Viscosity

Due to the high viscosity of the rubber compound, it was necessary to use a parallel plate plastometer to measure the shear viscosity. The model of parallel plate plastometer used is the MZ-4014. The measurement was made at 23 ± 2 °C. The measurement principle is that, under a given temperature, for a squeezing flow with constant volume, the shear viscosity η can be calculated with the following formula using applied load $F_{N}$, load time *t*, measured sample heights of $h_{0}$ and h before and after the load $F_{N}$, and measured sample volume $V_{r}$ [47]:

(37) $$\frac{F_{N}t}{3\eta V_{r}}=(\frac{1}{h}-\frac{1}{h_{0}})+\frac{V_{r}}{8\pi }(\frac{1}{h^{4}}-\frac{1}{h_{0}{}^{4}})$$

The sample was a cylindrical sample with a diameter of 16 mm and a thickness of 3 mm. The applied load was 49 N, and the applied load time was 60 s. For the above three rubber compounds, the shear viscosity values obtained by calculation are shown in Table 2.

Since the temperature changes from 23 °C to 50 °C during the experiment in previous studies, the viscosity corresponding to the temperature change can calculate according to the theoretical formula.

(2) Sliding friction coefficient

The sliding friction coefficient of the rubber compounds at room temperature was measured by Anton Paar TRB^3^, and the measurement was made at 23 ± 2 °C. To obtain the approximate value of the sliding friction coefficient between the rubber strip and the inner wall of the barrel, the measured linear speed was made close to the linear speed of the disc rotation squeezing model, and the unit area pressure applied to the test metal element close to the average pressure in the disc rotation squeezing model. The test metal element material is Q235-A. The values of the sliding friction coefficient obtained by measurement are shown in Table 3.

## 5. Results and Discussion

(1) Pressure distribution

In Section 2.2, according to Equation (6), the average squeezing velocity V of the model device in Table 1 at N = 30, 45, and 60 rpm was −11.4, −17.1, and −22.8 mm∙s^−1^, respectively. In Figure 11, the $h_{1}$(=H), $h_{2}$, and $h_{3}$ of the design wedge gap at the different positions of the pressure sensor installed in the upper (U), middle (M), and lower (L) sections were 5.4, 4.7, and 2.6 mm, respectively.

**Figure 12:**
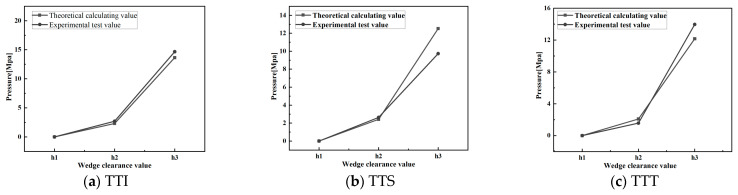
Comparison of calculated and experimental pressure distributions for TTI, TTS, and TTT at 30 rpm of the disc.

**Figure 13:**
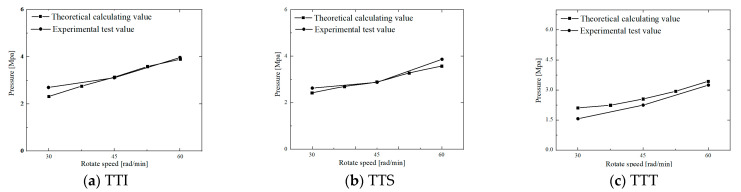
Comparison of calculated and experimental pressure changes of h2 = 4.7 mm for TTI, TTS, and TTT at different disc speeds.

The w value in Table 1, the η value of different rubber compounds in Table 2, the V value at different disc speeds, and the h value at different positions were substituted into Equation (12) ($\bar{p}=\eta Vw^{2}∕h^{3}$) to obtain the pressure distributions of different rubber compounds at different disc speeds. Figure 12 shows the pressure distribution of TTI, TTS, and TTT at N = 30 rpm. Figure 13 shows the pressure changes of TTI, TTS, and TTT in the position of $h_{2}=4.7$ mm at different disc speeds.

Figure 12 shows that, under a given disc speed, for different kinds of rubber strips, both theoretical prediction and experimental data had approximate pressure distribution curves, and the increase in pressure with the decrease in h in the region of large h was much slower than that in the region of small h, because $\bar{p}$ was proportional to the reciprocal of the third power of h (Equation (12)). However, except for the start position of squeezing $h_{1}$(=H), the difference between the theoretical predicted pressure and the experimental value is 0.5–2 Mpa. This is the result of two main factors: average squeezing velocity V and viscosity η. In the initial stage of squeezing, V is greater than the instantaneous squeezing velocity $\dot{h}$ (Figure 5), and the theoretical pressure obtained using Equation (12) is higher than the test data. In the middle and end stages of squeezing, V is less than $\dot{h}$ (Figure 5), and there are also obvious shear thinning and viscous heat generation effects. Although the pressure in the last two stages was calculated by using V, which was smaller than $\dot{h}$, the original viscosity data were larger, and the viscosity decrease caused by shear thinning and viscous heat generation was not considered; therefore, the theoretical prediction pressure was higher than that attained in the experimental data.

Figure 13 shows that both pressures obtained by theoretical calculation and experimental measurement increase almost linearly with increasing disc speed; the reason for this phenomenon is that $\bar{p}$ is proportional to the disc speed (Equations (6) and (13)). Additionally, the difference between theoretical pressure and the experimental value is about 0.05–0.52 Mpa. However, Figure 13 also shows that the theoretical value was greater than the experimental data, and the difference increased significantly with increasing N. This is because the position of $h_{2}=4.7$ mm occurs in the middle stage of squeezing. With the increase in disc speed N, the effects of shear thinning and viscous heat generation caused by the increase in shear rate (Equation (27)) increase. It has been reported in the literature [3] that the temperature of the squeezed TTI rubber compound is approximately 27 °C higher than room temperature when the disc rotation squeezing model test for TTI strips was carried out under N=60 rpm without cooling the disc and the semicylinder.

(2) Power consumption

The density of the rubber compound is approximately 1.5 × 103 kg·m^−3^. The relevant parameter values were substituted into Equation (37) to obtain the power consumptions of P1, P2, and P3 for the TTI, TTS, and TTT at different disc speeds. The range of P1 is 1.8 × 10^−6^~1.5 × 10^−5^ W, P2 is 2.5~13 W, and P3 is 200~900 W. Compared with P3, P1 and P2 were small and negligible. In other words, during the feeding process, the power consumption of the strip through the feed wedge clearance mainly comes from the sliding friction of the strip along the barrel. This conclusion is of great significance for the feed structure design of a cold-feed extruder.

**Figure 14:**
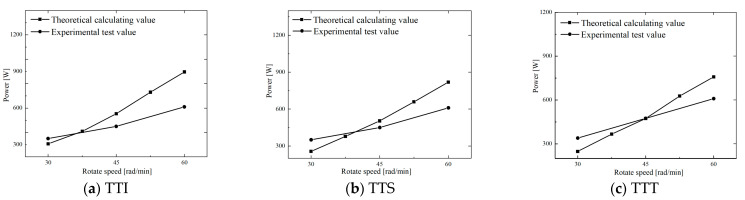
Comparison of calculated and experimental power consumptions for TTI, TTS, and TTT at different disc speeds.

Figure 14 shows the comparison of the calculated power (P3) and experimental power for TTI, TTS, and TTT at different disc speeds.

Figure 14 shows that the power consumption obtained by theoretical calculation and by experimental measurement increased linearly with increasing disc speed, and the former increased faster than the latter. The difference between the theoretical predicted pressure and the experimental value is about 40–200 W. Moreover, from the data comparison, it was found that the theoretical value is almost greater than the experimental value. This is mainly because the large viscosity data were used for the calculation of the total squeezing force without considering the significant decrease in viscosity caused by shear thinning and viscous heat generation, especially in the middle and end stages of the feeding process at a high disc speeds. In other words, during the test, with the increase in disc speed, the decrease in rubber viscosity would make the squeezing force small, eventually resulting in a decrease in power consumption. However, in the theoretical calculation, a constant and high value of rubber viscosity was used, so the theoretical value of the power consumption was greater than its experimental value.

However, in the actual feeding process of the cold feed extruder, due to the cooling of the screw and the feed barrel, the temperature rise in the rubber compound in the feed wedge clearance would be greatly limited. The effect of the decrease in viscosity caused by the temperature rise would be smaller, and the viscosity decrease mainly arises due to the contribution of shear thinning. This would increase the measured values of the pressure and power consumption, and lessen the difference between the theoretical prediction and the experimental data.

Therefore, for the feeding process of a cold feeding rubber extruder, the piecewise change in the squeezing velocity, shear thinning, viscous heat generation, and heat transfer must be considered if we are to develop a more accurate mathematical model. Since the Deborah number of the rubber compound passing through the feed wedge clearance at a high screw speed is probably large, feeding analysis theory should also consider the elastic response of the rubber compound [23,44,48].

## 6. Conclusions

This paper offered a theoretical analysis of the feeding process of rubber strips passing through a feed wedge clearance in a cold-feed extruder. First, by simplifying the screw flight rotation squeezing process into a disc rotation squeezing process, the instantaneous squeezing velocity $\dot{h}\left(t\right)$ in the disc rotation squeezing model was derived according to the feed wedge clearance geometry and the disc rotating speed. The $\dot{h}-t$ curve comprised two general parts: a constant acceleration squeeze in the early stage and a constant velocity squeeze in the later stage. To simplify the theoretical analysis, the squeezing process of the whole feed wedge clearance was regarded as constant velocity squeezing, and the average squeezing velocity V was used to replace the instantaneous squeezing velocity $\dot{h}$. Second, by transforming rotation squeezing into differential slab squeezing, mathematical expressions of the velocity distribution, pressure distribution, total squeezing force, and power consumption in the process of the rubber strip passing through the model wedge were derived; this analysis utilized the rectangular coordinate system under isothermal and quasi-steady assumptions and certain boundary conditions by using balance equations and the Newtonian viscous constitutive relation. Third, the shear viscosity and sliding friction coefficient of three kinds of rubber compounds were measured at room temperature and under conditions close to the actual feeding process by using a parallel plate plastometer and a CSM Anton Paar TRB^3^. The measured shear viscosity and sliding friction coefficient were substituted into the theoretical formulas to compare the theoretical prediction with previous experimental data. The comparison of pressure distribution and power consumption showed that the established theoretical model of the feeding process of cold-feed rubber extruders was not very accurate, on a quantitative level; however, it might reflect the main factors that affect the feeding process, and can reveal the main trends in feeding behavior. It was also noted that the piecewise change of the squeezing velocity, shear thinning, viscous heat generation, and heat transfer must be considered in a more accurate mathematical model, and the elastic response of the rubber compound should also be considered at a high screw speeds.

## Figures and Tables

**Table 1:** Geometric parameters of the model device used in this experiment.

D (mm)	w (mm)	R (mm)	$R_{1}\;(\mathrm{mm})$	a (mm)	b (mm)	H (mm)	$\theta_{0}\;({}^{\circ})$
65	10	32.5	32.5	2	5	5.4	85

**Table 2:** Viscosity values of different rubber compounds at room temperature.

Rubber Compound	TTI	TTS	TTT
Viscosity (MPa·s)	0.24	0.22	0.21

**Table 3:** Sliding friction coefficients of different rubber compounds at room temperature.

Rubber Compound	TTI	TTS	TTT
Sliding friction coefficient *	1.0 ± 0.015	0.9 ± 0.01	1.0 ± 0.021

* Average approximation at different disc speeds (30, 45, and 60 rpm) in a disc rotation squeezing model with D = 65 mm.

## References

[1] Schöppner V., Schadomsky M., Hopmann C., Lemke F. (2016). Investigations of the mixing behaviour of pin-type rubber extruders. AIP Conf. Proc..

[2] Lewandowski A., Wilczyński K. (2022). Modeling of Twin Screw Extrusion of Polymeric Materials. Polymers.

[3] Francis P.J.J., Joseph R., George K.E. (1997). Significance of Feeding Rate in the Extrusion of Filled and Gum IIR Vulcanizates. Int. J. Polym. Mater. Polym. Biomater..

[4] Water H. (1981). Schiesser and Zürich, Apparatus for Automatic Uniform Drawing-in of Elastomeric Material into Worm Extruders. U.S. Patent.

[5] Yanchang L., Fang Y., Chong M., Zhenglin H., Guangyi L. (2020). Feeding Behaviour of Cold-Feed Rubber Extruders. Polym. Mater. Sci. Eng..

[6] Jianbin L., Liangwei F., Jinbo Z., Shejun D. (1984). Study on the Conveying Capacity of Feed Section of Rubber Cold Feeding extruder. Rubber/Plast. Technol. Equip..

[7] Limper A., Schramm D. (2002). Process Description for the Extrusion of Rubber Compounds-Development and Evaluation of a Screw Design Software. Macromol. Mater. Eng..

[8] Alan N. (2012). Gent, Engineering with Rubber.

[9] Raymond L.C. (1988). Extruder for Elastomeric Material. U.S. Patent.

[10] Gerd C.L. (1991). Method of Maintaining the Force of a Stripper Blade on an Extruder Feed Roller Constant and an Apparatus therefor. U.S. Patent.

[11] Baiyuan L., Yanchang L., Penzhen L., Dianrui Z. (1999). Study on the influence of feeding method on extrusion process of rubber cold feeding extruder. Rubber Technol. Equip..

[12] John L.R. (2007). Leakage-free Feed Roll Assembly for an Extruder Machine. U.S. Patent.

[13] Thomas J.O. (2013). Extruder Feed Section with Pivotable Feed Roll Assembly. U.S. Patent.

[14] Brand W. (1974). Apparatus to Control Feed of Material to an Extruder. U.S. Patent.

[15] Ernest S. (1975). Ulm, Feed Control Mechanism. U.S. Patent.

[16] Anders D. (1981). Method of and Apparatus for the Controlled Feeding of Quantity of Material into the Intake Opeening of an Extruder for Processing Rubber or Plastics Material. U.S. Patent.

[17] Winther G., Almdal K., Kramer O. (1991). Determination of polymer melt viscosity by squeezing flow with constant plate velocity. J. Non-Newtonian Fluid Mech..

[18] Tashtoush B., Tahat M., Probert D. (2001). Heat transfers and radial flows via a visous fluid squeezed between two parallel disks. Appl. Energy.

[19] Debbaut B. (2001). Non-isothermal and viscoelastic effects in the squeeze flow between infinite plates. J. Non-Newton. Fluid Mech..

[20] Phan-Thien N., Tanner R. (1984). Lubrication squeeze-film theory for the oldroyd-b fluid. J. Non-Newton. Fluid Mech..

[21] Phan-Thien N., Dudek J., Boger D., Tirtaatmadja V. (1985). Squeeze film flow of ideal elastic liquids. J. Non-Newton. Fluid Mech..

[22] Gartling D.K., Phan-Thien N. (1984). A numerical simulation of a plastic fluid in a parallel-plate plastometer. J. Non-Newton. Fluid Mech..

[23] Phan-Thien N., Sugeng F., Tanner R. (1987). The squeeze-film flow of a viscoelastic fluid. J. Non-Newt. Fluid Mech..

[24] Lee S., Denn M., Crochet M., Metzner A., Riggins G. (1984). Compressive flow between parallel disks: II. oscillatory behavior of viscoelastic materials under a constant load. J. Non-Newton. Fluid Mech..

[25] Lipscomb G., Denn M. (1984). Flow of bingham fluids in complex geometries. J. Non-Newton. Fluid Mech..

[26] Kompani M., Venerus D.C. (2000). Equibiaxial extensional flow of polymer melts via lubricated squeezing flow. I. Experimental analysis. Rheol. Acta.

[27] Venerus D.C., Kompani M., Bernstein B. (2000). Equibiaxial extensional flow of polymer melts via lubricated squeezing flow. II. Flow modeling. Rheol. Acta.

[28] Jackson J.D. (1963). A study of squeezing flow. Flow Turbul. Combust..

[29] Smyrnaios D., Tsamopoulos J. (2001). Squeeze flow of Bingham plastics. J. Non-Newton. Fluid Mech..

[30] Alexandrou A.N., Florides G.C., Georgiou G.C. (2012). Squeeze Flow of Semi-Solid Slurries. Solid State Phenom..

[31] Muravleva L. (2015). Squeeze plane flow of viscoplastic Bingham material. J. Non-Newton. Fluid Mech..

[32] Muravleva L. (2017). Axisymmetric squeeze flow of a viscoplastic Bingham medium. J. Non-Newton. Fluid Mech..

[33] Muravleva L. (2020). Squeeze flow of Bingham, Casson and Herschel-Bulkley fluids with yield slip at the wall by accelerated augmented Lagrangian method. J. Non-Newton. Fluid Mech..

[34] Fusi L., Farina A., Rosso F. (2015). Planar squeeze flow of a bingham fluid. J. Non-Newton. Fluid Mech..

[35] Fusi L., Farina A., Rosso F. (2016). Squeeze flow of a Bingham-type fluid with elastic core. Int. J. Non-Newton. Fluid Mech..

[36] Sherwood J., Durban D. (1996). Squeeze flow of a power-law viscoplastic solid. J. Non-Newton. Fluid Mech..

[37] Sherwood J., Durban D. (1998). Squeeze-flow of a Herschel–Bulkley fluid. J. Non-Newton. Fluid Mech..

[38] Adams M., Aydin I., Briscoe B., Sinha S. (1997). A finite element analysis of the squeeze flow of an elasto-viscoplastic paste material. J. Non-Newton. Fluid Mech..

[39] Lawal A., Kalyon D.M. (1998). Squeezing flow of viscoplastic fluids subject to wall slip. Polym. Eng. Sci..

[40] Muravleva L. (2019). Axisymmetric squeeze flow of a Casson medium. J. Non-Newton. Fluid Mech..

[41] Kaushik P., Mondal P.K., Chakraborty S. (2016). Flow dynamics of a viscoelastic fluid squeezed and extruded between two parallel plates. J. Non-Newton. Fluid Mech..

[42] Phan-Thien N. (1990). Sliding and squeezing flow of a viscoelastic fluid in a wedge. Math. Und. Phys..

[43] Oliver D.R. (1979). The influence of fluid inertia, viscosity and extra stress on the load bearing capacity of a squeeze film of oil. Appl. Sci. Res..

[44] Engmann J., Servais C., Burbidge A.S. (2005). Squeeze flow theory and applications to rheometry: A review. J. Non-Newton. Fluid Mech..

[45] McClelland M.A., Finlayson B.A. (1983). Squeezing flow of elastic liquids. J. Non-Newton. Fluid Mech..

[46] Dienes G.J., Klemm H.F. (1946). Theory and Application of the Parallel Plate Plastometer. J. Appl. Phys..

[47] Gent A.N. (1960). Theory of the parallel plate viscometer. Br. J. Appl. Phys..

[48] Grimm R.J. (1978). Squeezing flows of polymeric liquids. AIChE J..

